# Use of putative hepatoprotective agents as an adjunct to anti-TB treatment in Europe

**DOI:** 10.5588/ijtldopen.24.0498

**Published:** 2025-02-01

**Authors:** O. Kirakosyan, M. Reimann, A.B. Andersen, A. Bjarnason, Á. Bakos, A.M. Dyrhol-Riise, A.M. McLaughlin, C. Nita, D. Pieridou, D. Chesov, E.V. Davidavičienė, G. Günther, H. Atshemyan, I. Muylle, I. Solovic, J. Bruchfeld, K. Manika, L. Kuksa, L.R. Codecasa, M. Stosic, M. Skowroński, M.J. Makek, M. Fréchet Jachym, M. Knappik, M. Santin, N. Yatskevich, O. Konstantynovska, O. Akkerman, P. Svetina, P. Viiklepp, R. Duarte, S. Zeynel, T. Togonidze, T. Vasankari, V. Parris, Ş. Özkara, C. Lange, T.T. Brehm

**Affiliations:** ^1^ Department of Clinical Infectious Diseases, Research Center Borstel, Leibniz Lung Center, Borstel, Germany;; ^2^ German Center for Infection Research (DZIF), Partner Site Hamburg-Lübeck-Borstel-Riems, Germany;; ^3^ Respiratory Medicine & International Health, University of Lübeck, Lübeck, Germany;; ^4^ Odense University Hospital, Department of Infectious Diseases, Copenhagen, Denmark;; ^5^ Faculty of Medicine, University of Iceland, Reykjavik, Iceland;; ^6^ Koranyi National Institute for Pulmonology, Budapest, Hungary;; ^7^ Department of Infectious Diseases, Oslo University Hospital, Oslo, Norway;; ^8^ University of Oslo, Oslo, Norway;; ^9^ National TB Centre, St James’s Hospital, Dublin, Ireland;; ^10^ Marius Nasta National Center of Pneumology, Bucharest, Romania;; ^11^ National Reference Laboratory for Mycobacteria, Nicosia General Hospital, Nicosia, Cyprus;; ^12^ Department of Pulmonology and Allergology, State University of Medicine and Pharmaceutics "Nicolae Testemiţanu", Chisinau, Moldova;; ^13^ Vilnius University Hospital Santaros Klinikos, Department of Tuberculosis State Information System, Vilnius, Lithuania;; ^14^ Department of Pulmonary Medicine and Allergology, Inselspital, Bern University Hospital, University of Bern, Bern, Switzerland;; ^15^ Department of Medical Sciences, School of Medicine, University of Namibia, Windhoek, Namibia;; ^16^ National Center of Pulmonology, Yerevan, Armenia;; ^17^ Division of Pneumology, Onze-Lieve-Vrouw Ziekenhuis (OLV) Aalst, Aalst, Belgium;; ^18^ National Institute for TB, Lung Diseases and Thoracic Surgery, Vysne Hagy, Slovakia;; ^19^ Unit of Infectious Diseases, Department of Medicine, Karolinska Institute, Stockholm, Sweden;; ^20^ Department of Infectious Diseases, Karolinska University Hospital, Stockholm, Sweden;; ^21^ Respiratory Diseases and Tuberculosis Pulmonary Department, Aristotle University of Thessaloniki, "G. Papanikolaou" Hospital, Thessaloniki, Greece;; ^22^ Riga East University Hospital, Tuberculosis and Lung Disease Clinic, WHO CC, Riga, Latvia;; ^23^ Regional TB Reference Centre, Villa Marelli Institute-Niguarda Hospital, Milan, Italy;; ^24^ Institute of Public Health of Serbia "Dr Milan Jovanovic Batut", Belgrade, Serbia;; ^25^ University of Health and Business Studies Valjevo, Valjevo, Serbia;; ^26^ Tuberculosis Department, Wielkopolskie Center of Pulmonology and Thoracic Surgery, Poznań, Poland;; ^27^ University of Zagreb, School of Medicine, Zagreb, Croatia;; ^28^ University Hospital Centre Zagreb, Department for Pulmonary Diseases, Zagreb, Croatia;; ^29^ Centre Hospitalier de Bligny, Briis-sous-Forges, France;; ^30^ Klinik Penzing, Vienna, Austria;; ^31^ Tuberculosis Unit, Department of Infectious Diseases, Bellvitge University Hospital-Bellvitge Biomedical Research Institute (IDIBELL), L’Hospitalet de Llobregat, Barcelona, Spain;; ^32^ Department of Clinical Sciences, University of Barcelona, L’Hospitalet de Llobregat, Barcelona, Spain;; ^33^ Centre for Biomedical Research in Infectious Diseases Network (CIBERINFEC), Instituto de Salud Carlos III, Madrid, Spain;; ^34^ The Republican Scientific and Practical Center for Pulmonology and Tuberculosis, Minsk, Belarus;; ^35^ V. N. Karazin Kharkiv National University, Department of Infectious Diseases and Clinical Immunology, Kharkiv, Ukraine;; ^36^ Regional Phtisiopulmonological Center of the Kharkiv Regional Council, Kharkiv, Ukraine;; ^37^ University of Groningen, University Medical Centrum Groningen, Dept of Pulmonary Diseases and Tuberculosis, Groningen, The Netherlands;; ^38^ University of Groningen, University Medical Centrum Groningen, TB Center Beatrixoord, Groningen, The Netherlands;; ^39^ National TB Program and Tuberculosis Registry of Republic of Slovenia, University Clinic of Respiratory and Allergic Diseases Golnik, Golnik, Slovenia;; ^40^ National Institute for Health Development, Tallinn, Estonia;; ^41^ Unidade de Investigação em Epidemiologia (EPI Unit), Instituto de Saúde Pública da Universidade do Porto, Porto, Portugal;; ^42^ Departamento de Saúde Comunitaria, Estudos de Populações, Instituto de Ciências Biomédicas Abel Salazar, Universidade do Porto, Porto, Portugal;; ^43^ Serviço de Pneumologia, Centro Hospitalar Vila Nova de Gaia/Espinho, Vila Nova de Gaia, Portugal;; ^44^ Unidade de Investigação Clínica, Administração Regional de Saúde do Norte, Porto, Portugal;; ^45^ Institute for Lung Diseases and Tuberculosis, Skopje, North Macedonia;; ^46^ National Center for Tuberculosis and Lung Diseases, Tbilisi, Georgia;; ^47^ Finnish Lung Health Association (Filha), Helsinki, Finland;; ^48^ University of Turku, Turku, Finland;; ^49^ London North West University Healthcare NHS Trust, London, UK;; ^50^ Atatürk Chest Diseases and Chest Surgery Education and Research Hospital, 8th Clinic, Sanatoryum Caddesi, Ankara, Turkey;; ^51^ Nergis Sokak 15/4, Ankara, Turkey;; ^52^ Baylor College of Medicine and Texas Children's Hospital, Global Tuberculosis Program, Houston, Texas, USA;; ^53^ Institute for Infection Research and Vaccine Development (IIRVD), University Medical Center Hamburg-Eppendorf, Hamburg, Germany;; ^54^ Division of Infectious Diseases, I. Department of Internal Medicine, University Medical Center Hamburg-Eppendorf, Hamburg, Germany.

**Keywords:** hepatotoxicity, drug-induced liver injury, hepatitis, silibinin, ursodeoxycholic acid

## Abstract

**BACKGROUND:**

Anecdotal information suggests that clinical practice regarding the use of putative hepatoprotective agents in TB treatment varies across countries in the WHO European Region.

**METHODS:**

Between November 2023 and May 2024, we conducted a standardised questionnaire survey on the use of putative hepatoprotective agents in patients receiving TB treatment among Tuberculosis Network European Trials Group (TBnet) representatives in countries in the WHO European Region.

**RESULTS:**

We received valid responses from 37 of 53 countries (69.8%), with 16 (43.2%) reporting regular use of putative hepatoprotective agents during anti-TB treatment. Half of these countries (*n* = 8) are part of the former Soviet Union. In five countries, these agents are recommended by national guidelines. The most commonly used hepatoprotective agents were silibin/silymarin (*n* = 9, 56.3%), ursodeoxycholic acid (*n* = 5, 31.3%), and soy phospholipids (*n* = 4, 25.0%). Treatment duration varied, with 56.3% (*n* = 9) using them for less than 1 month, 18.8% (*n* = 3) for 1–3 months, and 18.8% (*n* = 3) for 4–6 months.

**CONCLUSIONS:**

Putative hepatoprotective agents are widely used as an adjunct to TB treatment in the WHO European Region, particularly in the countries of the former Soviet Union, some of which have included them in their national guidelines.

TB remains a major public health challenge in Europe, with an estimated 229,000 cases in the year 2022.^1^ The standard treatment regimen for drug-susceptible TB is a 6-month course of isoniazid (INH), rifampicin (RIF), pyrazinamide (PZA) and ethambutol (EMB).^2^ This treatment regimen has been shown to have a cure rate of about 88%.^3^ However, some of the anti-TB drugs used can be associated with drug-induced liver injury (DILI), which is a long-standing problem in the treatment of TB. Hepatotoxicity can cause acute liver failure, significantly reduce drug adherence and ultimately lead to drug resistance and treatment failure. The incidence of DILI in patients receiving anti-TB drugs for latent infection or TB disease varies widely, ranging from 1% to 58% in the literature.^4–6^ This variability is likely due to differences in study populations and treatment regimens. PZA is associated with the highest risk of hepatotoxicity, with reported incidence rates ranging from 5% to 58%.^7–11^ The mechanisms underlying PZA hepatotoxicity are poorly understood, but it is thought to cause liver injury via metabolic intermediates such as 5-hydroxy-pyrazinoic acid.^12^ In patients treated with INH alone for latent infection, the incidence of significant hepatotoxicity is approximately 1%.^6^ INH can cause DILI either through immune responses triggered by the bioactivation of the acetylhydrazine metabolite or by direct activation of reactive metabolites that bind to and damage cellular macromolecules.^13^ RIF has been shown to cause hepatotoxicity in approximately 1% to 2% of patients.^5,14^ It may interfere with bilirubin excretion and increase the risk of INH-induced hepatotoxicity by inducing the enzyme INH hydrolase. Consequently, the risk of hepatotoxicity in patients receiving a combination of INH and RIF is approximately 2.6%.^15^ EMB has only been reported anecdotally to cause transient elevations in serum aminotransferase levels.^16^ In addition to these standard regimens, second-line anti-TB drugs such as fluoroquinolones, bedaquiline, carbapenems, para-aminosalicylic acid (PAS) and prothionamide, which are used to treat RIF-resistant/multidrug-resistant (RR/MDR-TB), pre-extensively drug-resistant (pre-XDR-TB), or extensively drug-resistant TB (XDR-TB), as well as patients who have experienced hepatotoxicity on standard regimens, may also cause liver injury. In this context, several putative hepatoprotective agents, including *N*-acetylcysteine,^17^ silibinin/silymarin,^18^ ursodeoxycholic acid (UDCA),^19^ glycyrrhizin,^20^ and L-carnitine,^21^ have been suggested to mitigate liver injury and thus represent potentially promising agents to minimise the adverse effects of anti-TB drugs and improve overall treatment success.^22^ While the benefits of such agents are the subject of a controversial debate, there is little information on whether and how often they are currently used in Europe. To address this, we conducted a survey of representatives of the Tuberculosis Network European Trials Group (TBnet) in countries in the WHO European Region countries, focusing on the use of putative hepatoprotective agents in patients receiving antimicrobial treatment for TB.

## METHODS

### Questionnaire

A questionnaire was developed and subsequently piloted with two TB experts, whose feedback was used to refine and finalise the questionnaire. The questionnaire covered the availability of national surveillance data on the incidence of hepatotoxicity during anti-TB treatment, stratified for patients with drug-susceptible and drug-resistant TB. If such national data were unavailable, respondents were asked to estimate the incidence of hepatotoxicity in their country. Respondents were also asked about practices, indications and national guidelines for the use of putative hepatoprotective agents, as well as economic aspects of the use of these agents, including insurance coverage and estimated costs to patients. No ethics approval was required for this study as it did not involve patient data.

### Data collection

The questionnaire was distributed by e-mail to TBnet representatives from 41 of the 53 countries in the WHO European Region between November 2023 and May 2024. Andorra, Azerbaijan, Bosnia-Herzegovina, Israel, Kazakhstan, Kyrgyzstan, Monaco, Montenegro, San Marino, Tajikistan, Turkmenistan and Uzbekistan were excluded because of their small populations or lack of contacts, respectively. All representatives were required to have sufficient knowledge of English to understand the questionnaire, to have access to their national protocols and guidelines, and to complete the questionnaire using these protocols and guidelines.

### Statistical analysis

All statistical analyses were performed in R v4.2.2. (‘High Sierra’; R Computing, Vienna, Austria) within the *tidyverse*.^23^ Continuous variables are presented as the median and interquartile range for non-normally distributed data. Categorical variables are presented as frequencies and percentages. Graphs and figures were generated using the *ggplot* package.^24^

## RESULTS

**Figure 1.**
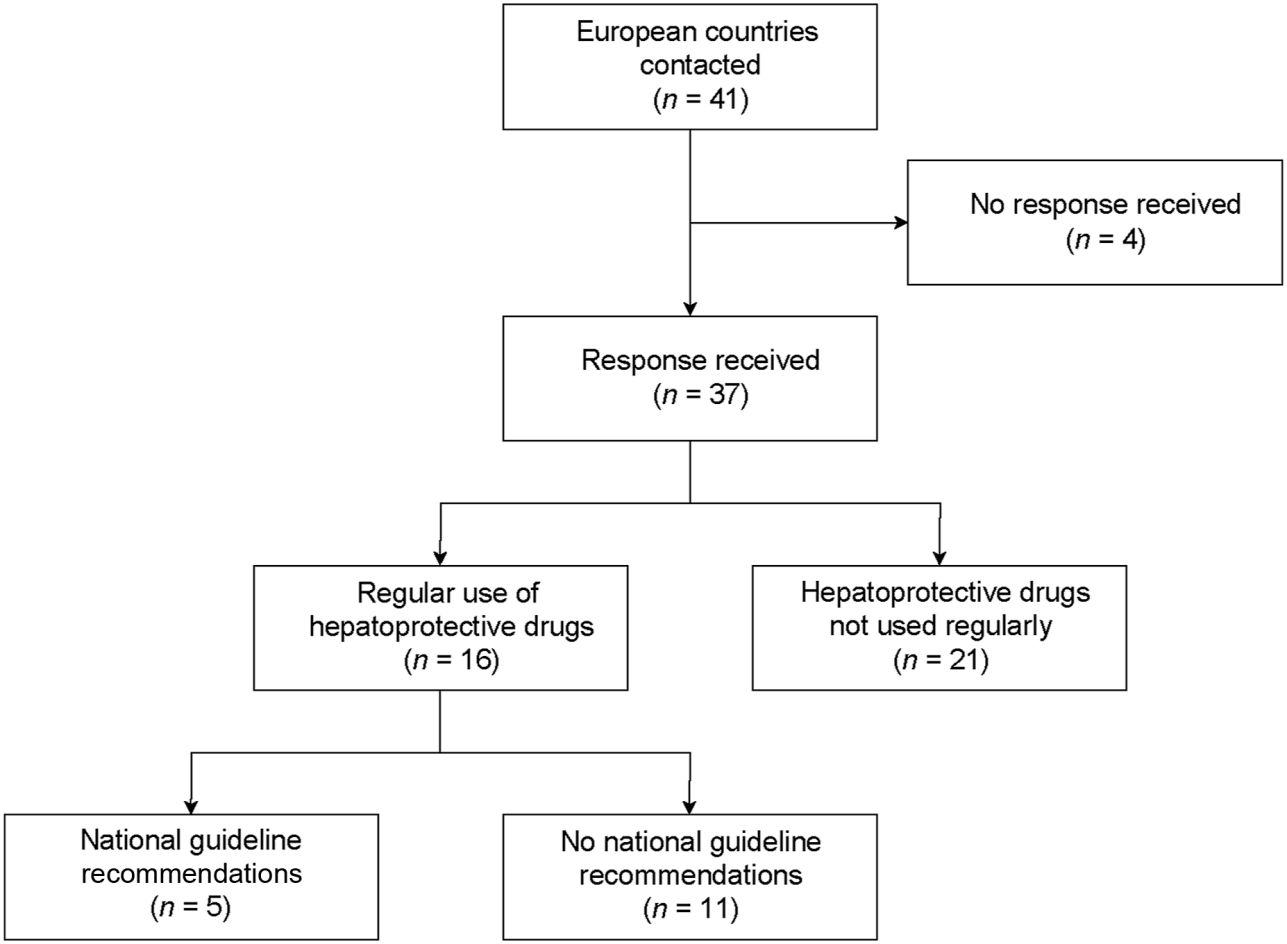
Flow diagram of participating countries

Responses were received from representatives of 37 out of the 41 countries that were contacted (90.2%) (Figure 1). Of these, only Slovenia was able to provide national data on the incidence of hepatotoxicity in patients receiving anti-TB treatment in the year 2020, reporting an incidence of 8%. Among countries without national data that estimated the incidence of hepatotoxicity for their country, the median estimated incidence was also 8.0% (interquartile range [IQR] 8.0–15.5%; *n* = 22 respondents) for all TB patients. The estimated rate of hepatotoxicity did not differ between patients with drug-susceptible TB (8.0%; IQR 8.0–15.5%; *n* = 22 respondents) and patients with drug-resistant TB (8.0%; IQR 2.5–15.5 %; *n* = 23 respondents).

**Figure 2.**
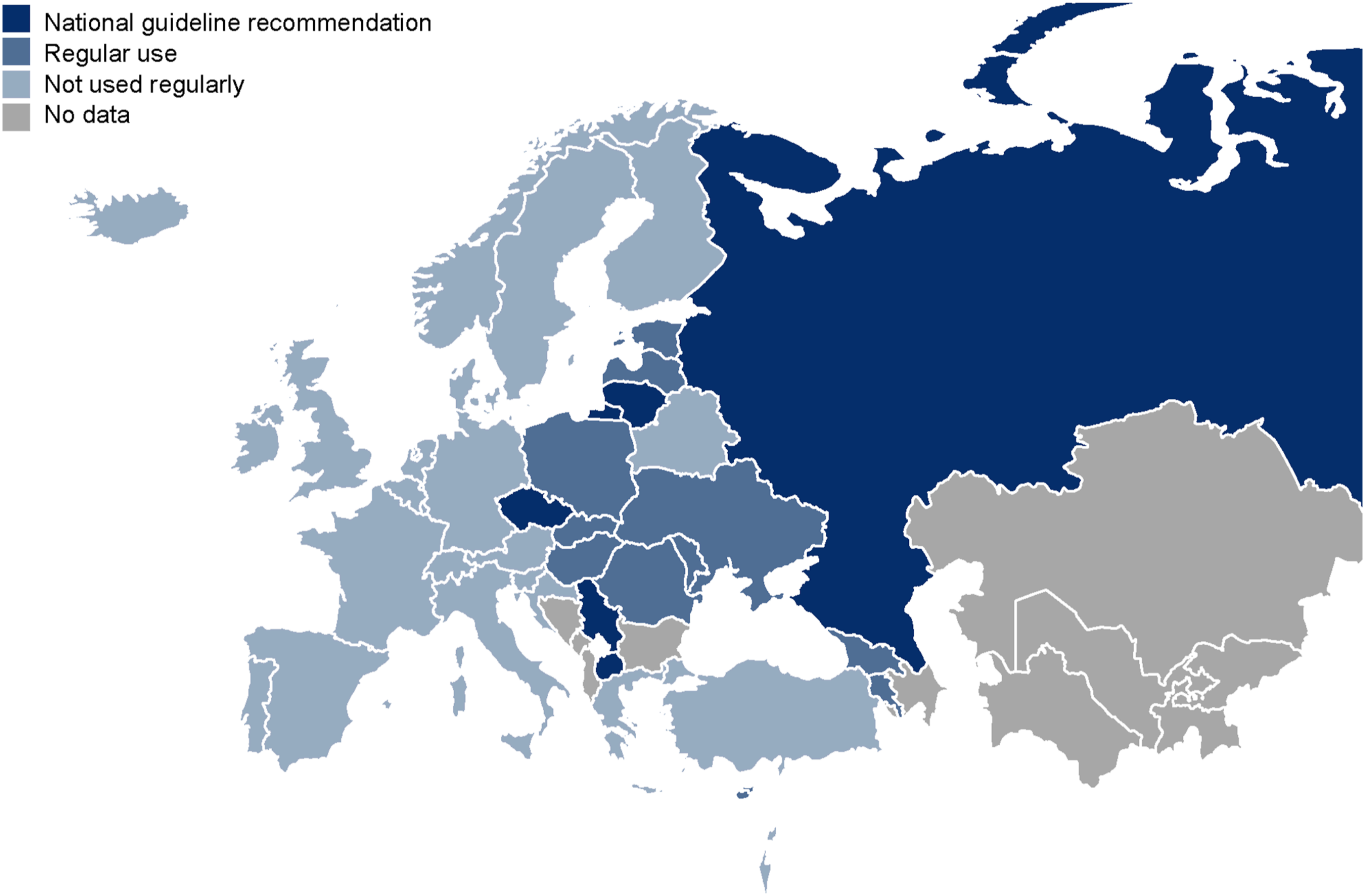
National practices on the use of putative hepatoprotective agents in TB treatment. Twelve countries were excluded because of their small population or lack of contacts through the TBnet network. A further four countries did not respond to the questionnaire.

Sixteen countries (43.2%) reported the regular use of putative hepatoprotective agents during anti-TB treatment. Half of these countries (*n* = 8) belong to the former Soviet Union. Five countries included them in national protocols or guideline recommendations (Figure 2). These agents were reported to be used for all patients throughout anti-TB treatment in three countries (3/16, 18.8%), only at the beginning of anti-TB treatment in seven countries (7/16, 43.8%), as a preventive measure only in the presence of risk factors in eight countries (8/16, 50.0%) and only when hepatotoxicity develops during anti-TB treatment in nine countries (9/16, 56.3%). The median estimated rate of hepatotoxicity during anti-TB treatment did not differ between countries that reported regular use of putative hepatoprotective agents and those that did not.

**Figure 3.**
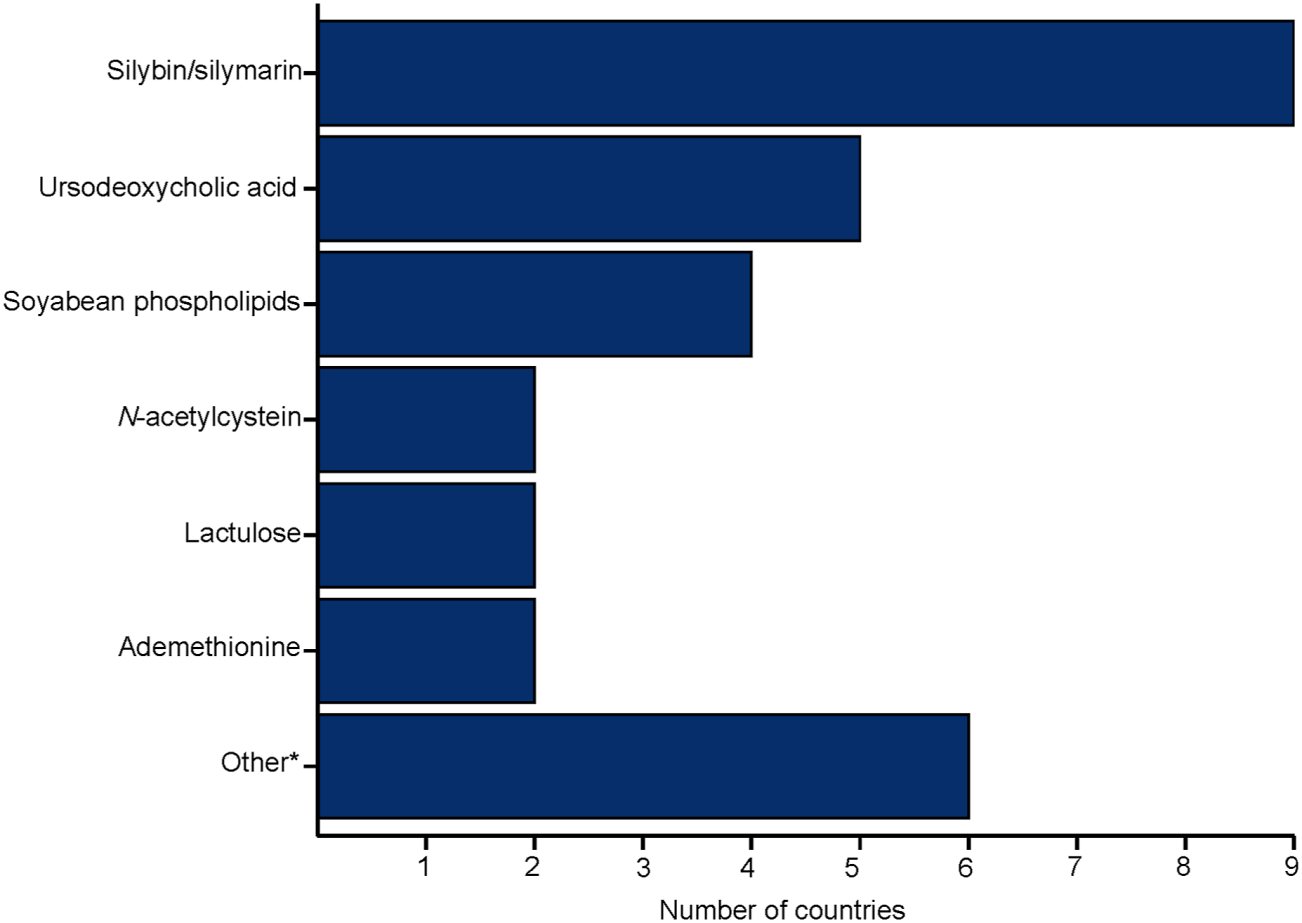
Putative hepatoprotective agents used as an adjunct to anti-TB treatment in European countries regularly or as part of guideline recommendations. *Includes ornithine, vitamin B, aspartate, Antral^®^ (Farmak JSC, Kyiv, Ukraine), arginine glutamate and Remaxol^®^ (POLYSAN Ltd, St Petersburg, Russia) (*n* = 1 country each).

The prescription of putative hepatoprotective agents was reported to be associated with the presence of hepatitis C virus (HCV) or hepatitis B virus (HBV) infection in 10 countries (10/16, 62.5%) and with HIV and alcohol consumption, in nine countries (9/16, 56.3%), respectively. The most common duration of adjunctive treatment with these agents was between less than 1 month (56.3%, 9/16), 1–3 months (3/16, 18.8%) and 4–6 months (3/16, 18.8%), while one country did not provide data (1/16, 6.3%). The most commonly used agents were silibinin/silymarin (9/16, 56.3%), UDCA (5/16, 31.3%), soy phospholipids (4/16, 25.0%), *N*-acetylcysteine (2/16, 12.5%), lactulose (2/16, 12.5%) and ademetionine (2/16, 12.5%) (Figure 3). Other agents mentioned by one country each were ornithine, vitamin B, aspartate, Antral^®^ (Farmak JSC, Kyiv, Ukraine) , arginine glutamate, and Remaxol^®^ (POLYSAN Ltd, St Petersburg, Russia). Twelve countries (12/16, 75%) reported financial coverage for these agents, but only for hospitalised patients in all but one country. The median average monthly cost of outpatient treatment in the eight countries providing this information was 10€ (IQR 9–15).

## DISCUSSION

This survey is the first to examine current practice and guideline recommendations for the use of putative hepatoprotective agents as an adjunct to anti-TB treatment in countries in the WHO European Region.

The incidence of DILI in TB patients has been reported in the published literature to range from 1% to 58%, reflecting differences in study populations, study settings and treatment regimens.^4–6^ There is also a lack of systematic surveillance data, which contributes to the uncertainty and variability of these estimates. Of the countries that were contacted in this study, only Slovenia was able to provide national data on the incidence of hepatotoxicity in patients receiving anti-TB treatment, reporting an incidence rate of 8%. Study participants were therefore asked to provide an estimate of the rate of hepatotoxicity during anti-TB treatment in their country, which was also 8%, and did not differ between drug-sensitive and drug-resistant TB patients. This median estimate is consistent with the results of previous studies and underlines the significant burden and clinical importance of hepatotoxicity during anti-TB treatment. The substantial rates of hepatotoxicity highlight the need for effective monitoring and management strategies to reduce adverse effects and ensure the success of anti-TB treatment regimens.

Interestingly, about half of the European countries participating in the survey reported the regular use of putative hepatoprotective agents during anti-TB treatment, with five countries even including them in national treatment guidelines. This finding is particularly noteworthy given the unclear evidence for the effectiveness of these agents in preventing DILI during anti-TB treatment. There are marked regional differences in the use of putative hepatoprotective agents in Europe. They are rarely used in Western Europe, whereas their use appears to be widespread in Eastern Europe. Notably, half of the countries reporting regular use of these agents (*n* = 8) are countries of the former Soviet Union. Traditional habits and beliefs appear to play an important role in assessing the efficacy of these agents. This survey was not designed to address differences in the incidence of hepatotoxicity during anti-TB treatment in countries where putative hepatoprotective agents are or are not used. Although the estimated incidences did not differ in these countries and did not support the use of these agents as an adjunct to anti-TB therapy, the quality of the estimated data does not provide sufficient evidence.

A retrospective study from Japan that analysed 389 patients with anti-TB drug-induced hepatotoxicity showed no difference between the use of the putative hepatoprotective agents UDCA, stronger neo-minophagen C or glycyrrhizin and the time to normalisation of liver enzymes, regardless of the severity of hepatotoxicity.^25^ On the other hand, a meta-analysis of 18 trials involving 3589 patients showed that the use of putative hepatoprotective agents contributed to an overall lower incidence of liver injury and fewer drug withdrawals compared with conventional anti-TB treatment alone.^26^ Another systematic review and network meta-analysis of 3,423 patients from 14 randomised controlled trials suggested that turmeric plus *Tinospora cordifolia*, *N*-acetylcysteine and a polyherbal product may be beneficial in preventing hepatotoxicity in patients receiving anti-TB treatment.^27^ However, these meta-analyses are based on a limited number of relatively small studies. Therefore, larger and more comprehensive prospective randomised controlled trials are needed to provide robust evidence on the potential benefit of different putative hepatoprotective agents as an adjunct to anti-TB treatment. Importantly, putative hepatoprotective agents may themselves cause liver damage and thus potentially harm TB patients.^28^ In addition, putative hepatoprotective agents are sometimes combined with additional herbal extracts that may pose a risk of drug-drug interactions when used with RIF-based anti-TB regimens. Also, one of the agents with potential hepatoprotective efficacy in the systematic review systematic review and network meta-analysis by Akkahadsee et al.^27^ is a polyherbal product containing several herbal extracts with unconfirmed interaction profiles.^29^ Herbal supplements can inhibit cytochrome enzymes, particularly *CYP3A4*, and potentially reduce RIF levels through metabolic interference, leading to subtherapeutic concentrations and impaired anti-TB efficacy. Notably, the above meta-analyses on the effectiveness of hepatoprotective agents in reducing hepatotoxicity do not evaluate their impact on TB treatment outcomes.

Previously identified risk factors associated with DILI during anti-TB treatment include age over 35 years, alcohol consumption, pre-existing liver disease, slow acetylator phenotype, malnutrition and viral hepatitis.^6^ Consistent with this, about half of the countries responding to the survey reported using putative hepatoprotective agents as a preventive measure only in the presence of such risk factors, including hepatitis B, hepatitis C, HIV, or alcohol use.

While the majority of countries reported financial coverage of putative hepatoprotective agents for hospitalised patients, patients are usually responsible for the cost of outpatient treatment. This discrepancy between free provision in hospitals and out-of-pocket expenses for outpatient treatment creates a financial barrier that may hinder adherence to these supportive therapies. With a median average monthly cost of treatment of 10€, the prophylactic use of these agents may represent an additional economic burden for some patients and may thus negatively affect compliance with anti-TB treatment.^30^ However, data on out-of-pocket costs for putative hepatoprotective agents were unavailable for many countries, and actual expenditures may be even higher in some settings.

This study has important limitations. First, because some countries were excluded because of their small population or lack of contacts, and because we received responses from only 37 of the 41 countries that were contacted, we do not have information on current practices and guideline recommendations for all countries in the WHO European Region. Second, although we asked respondents to answer all questions about current national practices, protocols and guideline recommendations, practices may vary between physicians, centres or regions within a country, so the results may not always be representative of the country. Third, national data on the incidence of hepatotoxicity were only available for one country, and the remaining countries were asked to estimate the incidence of hepatotoxicity for drug-sensitive and drug-resistant TB in their countries. Although the results are consistent with previous literature, these more subjective data may negatively affect the overall validity of the study. Finally, only eight of the 16 countries that regularly prescribe putative hepatoprotective agents provided information on the monthly treatment costs, which limits the generalisability of these results.

## CONCLUSIONS

Putative hepatoprotective agents are widely used in the WHO European Region, particularly in the countries of the former Soviet Union, as an adjunct to anti-TB therapy, and some countries have included them in their national policies and guidelines. However, there is a considerable need for further research into the potential role of different agents as an adjunct to anti-TB treatment in reducing liver toxicity and improving treatment outcomes.
